# Adiponectin and Its Receptors in the Ovary: Further Evidence for a Link between Obesity and Hyperandrogenism in Polycystic Ovary Syndrome 

**DOI:** 10.1371/journal.pone.0080416

**Published:** 2013-11-18

**Authors:** Fabio V. Comim, Kate Hardy, Stephen Franks

**Affiliations:** 1 Institute of Reproductive and Developmental Biology, Imperial College London, London, United Kingdom; 2 Federal University of Santa Maria (UFSM), Santa Maria, Brazil; VU University Medical Center, Netherlands

## Abstract

Polycystic ovary syndrome (PCOS), characterized by ovarian androgen excess, is the commonest endocrine disorder in women. Obesity increases androgen synthesis, a phenomenon attributed to the accompanying hyperinsulinemia. Our hypothesis was that adipokines, fat cell-derived hormones, play a direct role in modulating ovarian androgen secretion. Therefore, the aims of this study were to explore the effects of adipokines (in particular, adiponectin) on ovarian steroidogenesis and compare the expression of adiponectin receptors in ovaries from women with and without PCO. Sections of archived human ovaries (nine from women with normal ovaries and 16 with PCOS, classified histologically, with reference to menstrual history and ultrasound) were analysed by quantitative morphometry and the proportion of positive-labelling cells compared. In addition, studies of androgen production in relation to adipokine function in primary bovine theca cell culture were also performed. A significantly lower proportion of theca cells expressed adiponectin receptors 1 and 2 (AdipoR1, AdipoR2) in polycystic ovaries than in normal ovaries. In cultured theca cells, adiponectin suppressed androstenedione production and gene expression of LH receptor and key enzymes in the androgen synthesis pathway. Moreover, knockdown of genes for AdipoR1 and AdipoR2 was associated with increased androstenedione secretion by bovine theca cells. These results provide evidence for a direct link between fat cell metabolism and ovarian steroidogenesis, suggesting that disruption of adiponectin and/or its receptors plays a key role in pathogenesis of hyperandrogenism in PCOS.

## Introduction

Polycystic ovary syndrome (PCOS) is the commonest endocrine disorder in women, affecting 5-10% of females of reproductive age [1,2]. Hyperandrogenism is the hallmark of PCOS and originates predominantly from ovarian theca-interstitial cells, which are characterised by an increased capacity for androgen biosynthesis [3-6].

 Androgen production is markedly increased in PCOS in the presence of obesity, which has been attributed, at least in part, to the effect of higher than normal insulin levels on theca cell function [7]. However, a possible role for cytokine products of fat (adipokines) as a link between reproductive and metabolic abnormalities has been mooted [8]. In this regard, quantitatively and functionally, adiponectin (a 30kd protein) is considered to be one of the most important adipokines in human physiology [9]. It differs from most other adipokines by having what appears to be a protective effect on development of obesity. It has insulin sensitising and fat-burning properties that are similar to leptin but it has also been shown to have anti-oxidant and anti-atherogenic effects [9,10]. Its serum levels are inversely related to fat mass and are positively associated with lower risk of type 2 diabetes [9,11]. Although abnormal serum levels of various adipokines in PCOS have been reported [12-19] few studies have addressed the effects of adipokines on reproductive tissues [20,21]. 

Adiponectin levels vary little during the menstrual cycle [22]. Nevertheless a role for adiponectin has been suggested in oocyte maturation, granulosa cell proliferation and death, and as a modulator of estradiol and progesterone secretion [8,21,23,24]. Little is known, about its effects on steroidogenesis in theca cells but Spicer and colleagues observed that addition of adiponectin to primary bovine theca cells (TCs) cultured in the presence of luteinizing hormone (LH) and insulin, decreased androstenedione and progesterone production [20,25]. To date, these preliminary findings have been neither confirmed nor contradicted. Adiponectin receptors have been partially characterised in granulosa-lutein cells obtained from women undergoing IVF [8], but there have been no systematic studies in ovaries from women with PCOS.

In the present study, we demonstrate a decrease in the expression of adiponectin receptors in theca cells from polycystic ovaries compared with theca from normal ovaries. Furthermore, we show by means of suppression of gene expression by small interfering RNA (siRNA), that adiponectin, the principal adipokine in humans, decreases the production of androgens in theca cells *in vitro*. We therefore propose a novel mechanism of interaction between fat cell metabolism and steroidogenesis in PCOS that may help to explain why hyperandrogenism is more severe in obese and overweight than in lean women with PCOS.

## Materials and Methods

### Analysis of human ovaries

Sections of archived human ovaries (fixed in formalin and embedded in paraffin) collected between 1993 and 2003 from patients less than 50 years of age with benign gynecological disease were obtained from the Histopathology Bank of St. Mary’s Hospital (Imperial College Healthcare NHS Trust) in London, as previously described [26,27]. Overall, excluding the specimens considered unclassified or having concomitant pathology, 25 samples, 16 PCO and nine control ovaries, were available for analysis. Of the 16 women with PCOS, 11 had evidence of regular ovulatory cycles and five had oligomenorrhea or irregular menses (more extensive clinical details are given in Table S1). All specimens were collected with previous informed consent from patients and in conformity with the Human Tissue Act and the local Research Ethics Committee (2002/6400). In addition to histological information, clinical data, including menstrual cycle history and ultrasonography of the ovaries, were obtained (see Table S1). These data were used to establish a clear diagnosis of PCOS [28].

### Sample preparation and immunohistochemistry and analysis of images

Staining of all slides was carried out simultaneously in a single session. Briefly, slides were dewaxed and rehydrated through an alcohol series. Antigen was retrieved with citrate buffer (0.01 mol/litre, pH 6, boiling for 20 min), and exogenous peroxidases were blocked with 0.3% hydrogen peroxide in methanol (30 min). Nonspecific antibody-binding was reduced by 20 min incubation with 10% (vol/vol) normal goat serum in PBS (Invitrogen Life Technologies, Paisley, UK) supplemented with 4% (wt/vol) BSA (Sigma-Aldrich, Poole, UK). Sections were incubated overnight with antibodies to adiponectin receptor 1 (H-001-45) and adiponectin receptor 2 (H-001-23), both from Phoenix Pharmaceuticals, Burlingame,USA. The optimal dilutions of antibodies (both at 1:200) in 10% serum in PBS were determined by antibody dilution curves, as previously described [26,27]. After wash, a secondary antibody (goat anti-rabbit E0432, DAKO, Denmark at dilution of 1:200) was employed and then incubated with peroxidase-conjugated avidin-biotin complex (RTU-ABC Kit, Vector, Peterborough, UK); sections were exposed to HRP-conjugated 3,3’- diaminobenzidine tetrahydrochloride (DAB) to permit visualization of labelling. Slides were counterstained with haematoxylin (BDH, Poole, UK), re-hydrated in alcohol (70%, 95%, 100%) and mounted in DPX after 2 changes (5 min) in Histoclear®. Sections examined after replacement of the primary antibody by non-immune serum served as negative controls. After labelling, images of the theca cell layer observed in two arbitrarily chosen fields were taken, employing a DXM 1200 digital camera Nikon at 60X magnification. The proportions of positive theca cells were then calculated and recorded using the Lucia image analysis program (Nikon, Kingston-upon-Thames, UK). 

### Primary culture of bovine theca cells

Ovaries from adult non-pregnant cattle were obtained from an UK slaughterhouse (Anglo Dutch farms, Kent, UK). They were collected and transported in warm medium (DMEM/F12 with Penicillin, 100 μg/ml and Streptomycin, 0.5 μg/ml at 38°C) to the laboratory, where the ovaries were dissected and large antral follicles isolated. Theca cell isolation and culture procedures were performed as previously described by Stewart et al [20,29](Figure S1). Human recombinant adiponectin used in these experiments (ALX-522-063, Enzo Life Sciences,USA) was composed by high molecular and hexameric species. Briefly, theca cells were plated at 38.5 °C and 5% CO_2_ at a density of 1.5 × 10^5^ cells per 1.9 cm^2^. Subsequently, the D-MEM/F12 medium containing 1μg/ml Transferrin, 1 ng/ml Selenium, 1 μg/ml Linoleic acid, 100 UI/ml Penicilin, 100 μg/ml Streptomycin, 0.5 μg/ml Gentamicin) was changed, and cells were treated for 24 h with various concentrations of the adipokines resistin, visfatin, leptin (30, 300, and 600 ng/mL). The concentrations of adiponectin used were based on doses used in previous studies of theca cells in culture [20] and on reported serum levels of adiponectin [9,16,30]. For other adiponectin and other adipokines, the aim was use doses that span a range of serum concentrations from physiological to supraphysiological [13-16,30] . At the end of the experiment, medium was collected and androstenedione concentrations were measured using ELISA. Androstenedione concentrations were adjusted for the number of viable cells per well using a luminescent cell viability assay (Cell-TiterGlo, Promega, Madison,USA). 

### Adiponectin receptor gene knockdown, qPCR, and RT-PCR

The knockdown of the genes encoding proteins ADIPOR1 and ADIPOR2 or their common mediator, an adaptor protein containing a pleckstrin homology domain (APPL1), was carried out by using small-interfering RNA (siRNA) (DharmaconAccel^®^ siRNA; Dharmacon, Thermo Scientific Inc., Europe). In summary, bovine TCs were plated at a density of 1.5 × 10^4^ cells per 0.14 cm^2^ and incubated for 72 h with 1 nM siRNA specific for the respective targets (ADIPOR1, ADIPOR2, or APPl-1) and, as controls, scrambled siRNA, or medium without siRNA containing 10 ng/mL LH (as detailed in Table S2). Successful transfection (greater than 95%) was ascertained by detection of fluorescently-labeled scrambled siRNA within cells by microscopy. Medium was then removed and replaced with fresh medium with or without adiponectin (3 μg/mL). The samples were incubated at 38.5 °C for an additional 24 h, when the experiment was ended. Knockdowns with an efficiency higher than 55%, assessed by quantitative gene expression, were considered for evaluation of the effects on steroid production. Androstenedione levels in medium were adjusted for the number of viable cells in culture using the CellTiter-Glo^®^ Luminescent Cell Viability Assay (Promega, USA). Isolation of mRNA was prepared from the cellular extracts using Trizol^®^ (Invitrogen, Life Technologies Co, Paisley, UK) according to the manufacturer´s instructions. For smaller samples developing in growing surfaces less than 0.14 cm^2^ (96 well-plate chamber), a cell lysis buffer (RLT) from RNeasy^®^ Mini Kit (Qiagen, Texas, USA) was employed. Complementary DNA (cDNA) synthesis was obtained after treatment with DNAse I and SuperScript II reverse transcriptase (both from Invitrogen, Life Technologies Co, Paisley, UK). Details of the primers used and qPCR and RT-PCR reactions performed are provided in Table S3.

### Statistical analysis

Where appropriate, anonymised data were stored in Microsoft Excel files. Statistical analysis was performed using the software Instat3 (Graphpad Software, San Diego,USA). Differences between groups were tested using the Student’s t-test (if normal distribution) or Mann-Whitney test (if asymmetrical distribution). For the comparison of more than two groups with a normal distribution, one-way analysis of variance (ANOVA) and an additional post-test (Tukey’s or Dunnet’s test) were used; for data with an asymmetrical distribution, the Kruskal-Wallis test and Dunn’s multiple comparison test were used. In all cases, a probability value (p) less than 0.05 was considered statistically significant. Normally distributed data are represented by mean plus SEM or SD. 

## Results

### Reduced expression of adiponectin receptors (AdipoR1 and AdipoR2) in theca cells from polycystic ovaries

There was no significant difference between women with or without PCO in age (mean [SEM] PCO 33.7 [1.1] vs normal 33.2 [1.2] yrs) or BMI (26.8 [2.0] vs 22.6 [1.7] kg/m^2^). Only 3 women in this series (all with anovPCO) were obese (BMI >30) and four others (1 normal, 3 ovPCO) were overweight (BMI >25, <30) but the difference in BMI between anovPCO and ovPCO subjects was not significant). Comprehensive clinical details of all 25 subjects are shown in Table S1.

**Figure 1:**
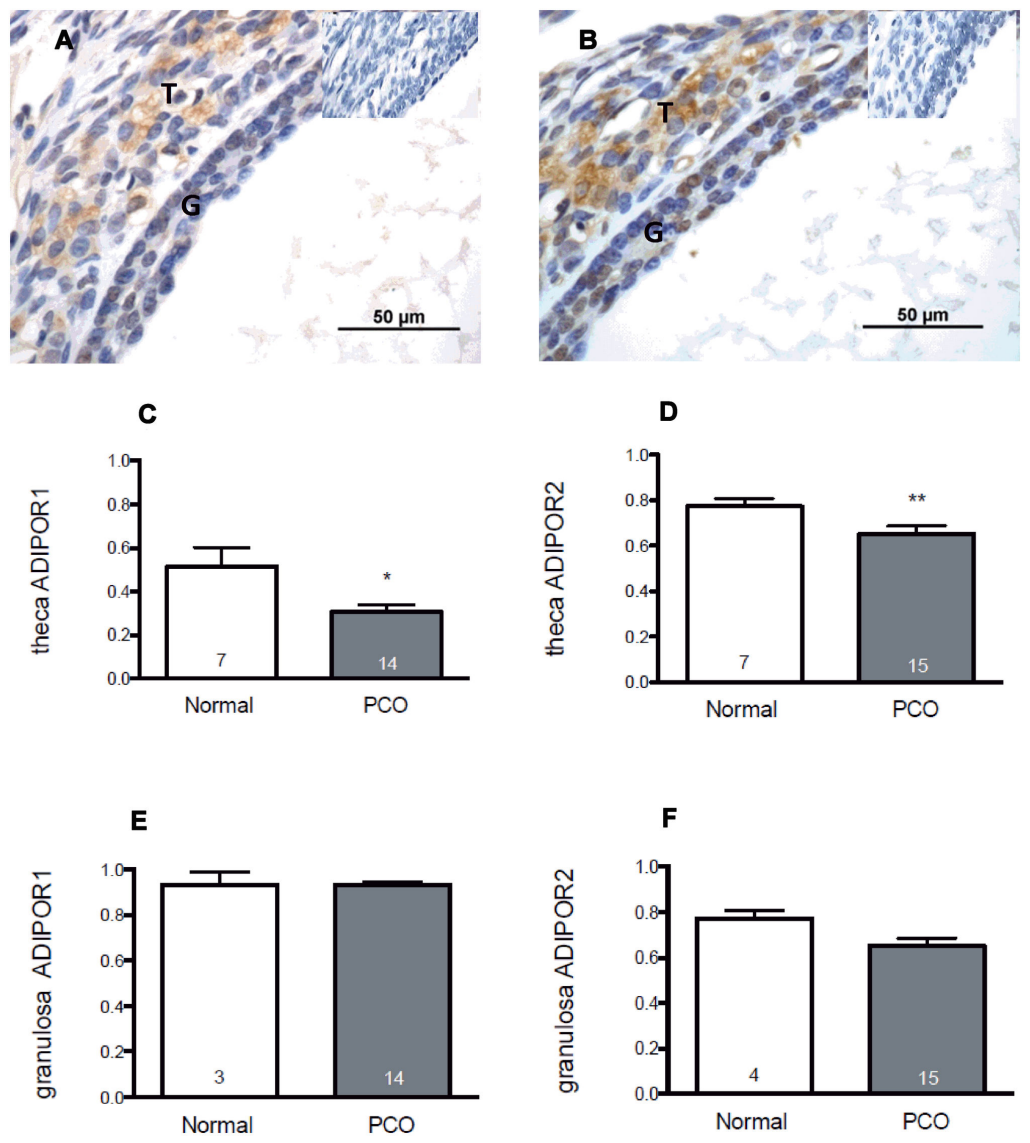
Expression of adiponectin receptors in follicles from normal and polycystic human ovaries. Both AdipoR1 (Figure 1a) and AdipoR2 (Figure 1b) were detected in granulosa (G) and theca (T) of antral follicles (insets show control [negative] sections). The proportions of theca and granulosa cells expressing protein for adiponectin receptor I & 2 (AdipoR1 and AdipoR2) in follicles from normal and polycystic ovaries are shown in the bar graphs. A total of 920 follicles were analysed. A significant reduction in the proportion of TCs labelled for (c) AdipoR1 and (d) AdipoR2 was demonstrated in polycystic ovaries compared with normal ovaries from healthy women (*p=0.002, **p=0.049, Mann-Whitney). No differences between PCO and control tissue were detected in the proportion of granulosa cells labelling for AdipoR1 (e) or AdipoR2 (f). Data shown are mean + SEM in up to 7 normal and 15 polycystic ovaries.

Overall, 726 follicles were examined for AdipoR1 staining and 920 follicles for AdipoR2 staining, including 85 antral follicles for AdipoR1 and 97 antral follicles for AdipoR2. Immunostaining for both AdipoR1 and AdipoR2 was present in both granulosa and theca cells (Figure 1a). In preantral follicles, receptor protein was detected in granulosa cells from the primary stage onwards in both normal and polycystic ovaries, and was similar in both types of ovary (data not shown). In antral follicles, adiponectin receptor expression was reduced in theca cells from polycystic ovaries compared with theca from normal ovaries. For AdipoR1, the proportion of positively stained TCs, mean (95% of confidence interval - CI) was 0.29 (0.20, 0.38) in polycystic ovaries versus 0.52 (0.32, 0.72) in normal ovaries (p = 0.002, Mann-Whitney test) (Figure 1b). In the case of AdipoR2, the proportion of immuno-positive theca cells was 0.65 (0.52, 0.78) in polycystic ovaries and 0.77 (0.69, 0.85) in the normal controls (p = 0.049; Mann-Whitney test) (Figure 1c). By contrast, no differences were noted between polycystic ovaries and normal ovaries in the proportions of granulosa cells (GCs) in antral follicles expressing AdipoR1 or AdipoR2. The proportion of GCs staining for AdipoR1 in polycystic ovaries (mean [95% of confidence interval - CI]) was 0.93 (0.90, 0.96), compared with 0.93 (0.68, 1.1) in normal ovaries (Figure 1c); for AdipoR2, the proportions were 0.77 (0.71, 0.82) and 0.85 (0.68, 1), respectively in PCO and control ovaries (p > 0.05) (Figure 1e). Because of the potential effect of obesity on adiponectin receptor expression we compared the proportions of follicles in women with PCO that expressed Adipo R1 and AdipoR2 in theca in women who were overweight or obese with expression in theca from lean PCO subjects. There was no significant difference in the proportion of Adipo R1 follicles between women who were overweight or obese and lean women overall or between lean and overweight/obese women with PCO (mean [SD] lean PCO, 0.33 [0.05] vs overweight PCO, 0.30 [0.05]. Similar results were observed with respect to Adipo R2 (lean PCO, 0.67 [0.02] vs overweight 0.67 [0.04]). 

### Adiponectin regulates androgen production by theca cells

**Figure 2:**
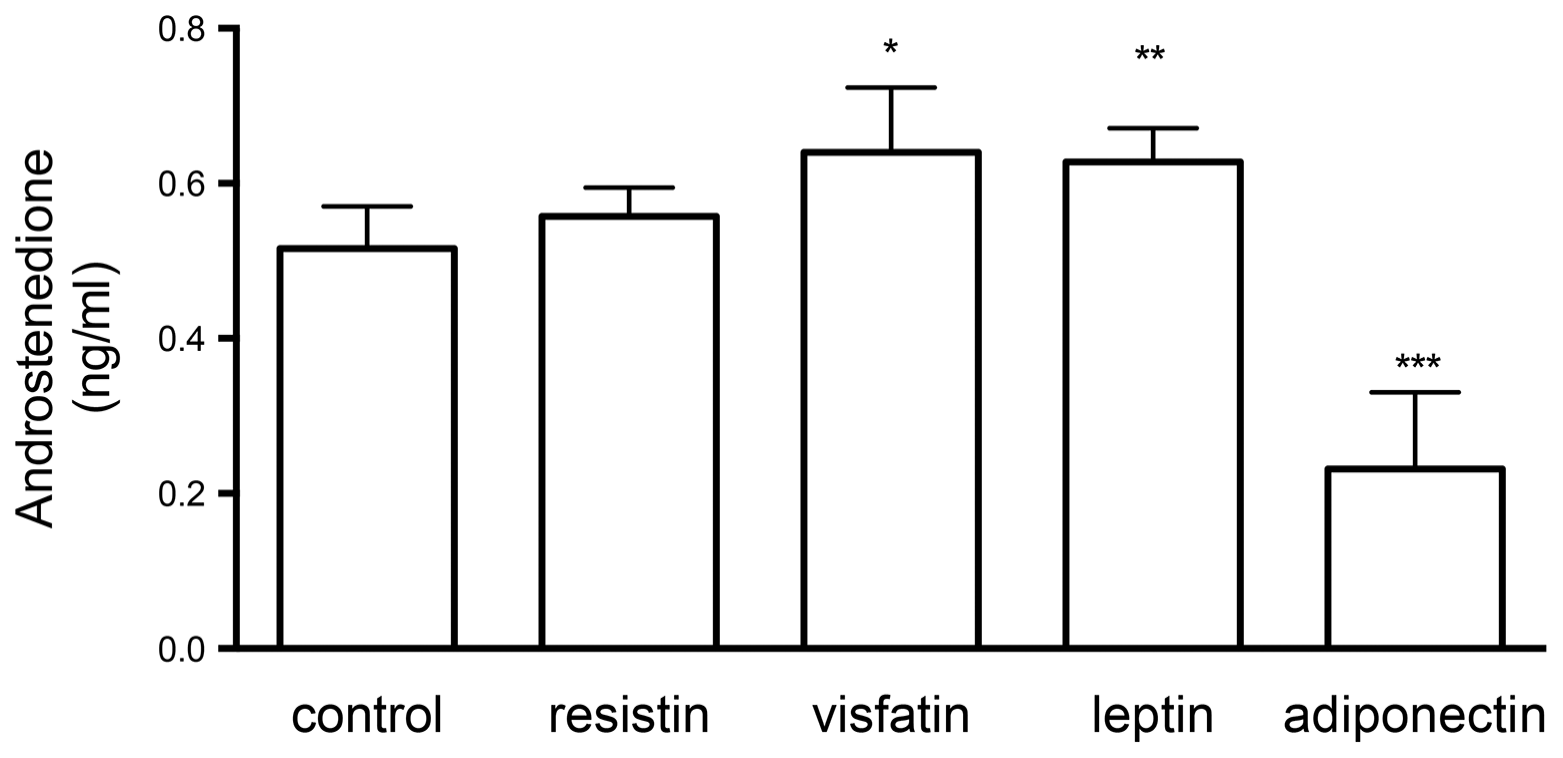
Effects of the adipokines resistin, visfatin, leptin, and adiponectin on androstenedione production *in* *vitro* in the presence of LH: compared to control cells, adiponectin treatment (3μg/mL) decreased levels of androstenedione in medium (****p= <0.0001) when bovine theca cells were treated with LH and insulin (both at 100 ng/mL). Small stimulatory effects of leptin and visfatin were also observed. The concentrations of adipokines were chosen on the basis of the relative serum levels in normal women. Data shown are mean+SD.

Incubation of TCs with adiponectin in the presence of LH and insulin resulted in a marked decrease in androstenedione levels in the medium, contrasting with a small but significant increase in the androstenedione levels observed with treatment with leptin and visfatin (but not resistin). In leptin- and visfatin-treated cells, the androstenedione levels in the medium were (mean ± SD) 0.63 ± 0.04 and 0.64 ± 0.08, respectively, both significantly higher than the levels found in control wells 0.54 ± 0.08 (p = 0.001; Kruskal-Wallis test and Dunn’s multiple comparison test)(Figure 2). The suppressive effect of adiponectin on androgen secretion was noted again in cells treated with LH in the absence of insulin, where the androstenedione levels (mean ± SD; 0.77 ± 0.11 ng/mL) were 49% of those noted in control cells (1.57 ± 0.26 ng/mL) (figure not shown). In the same experiment, neither leptin nor visfatin elicited a significant change in androgen production. 

**Figure 3:**
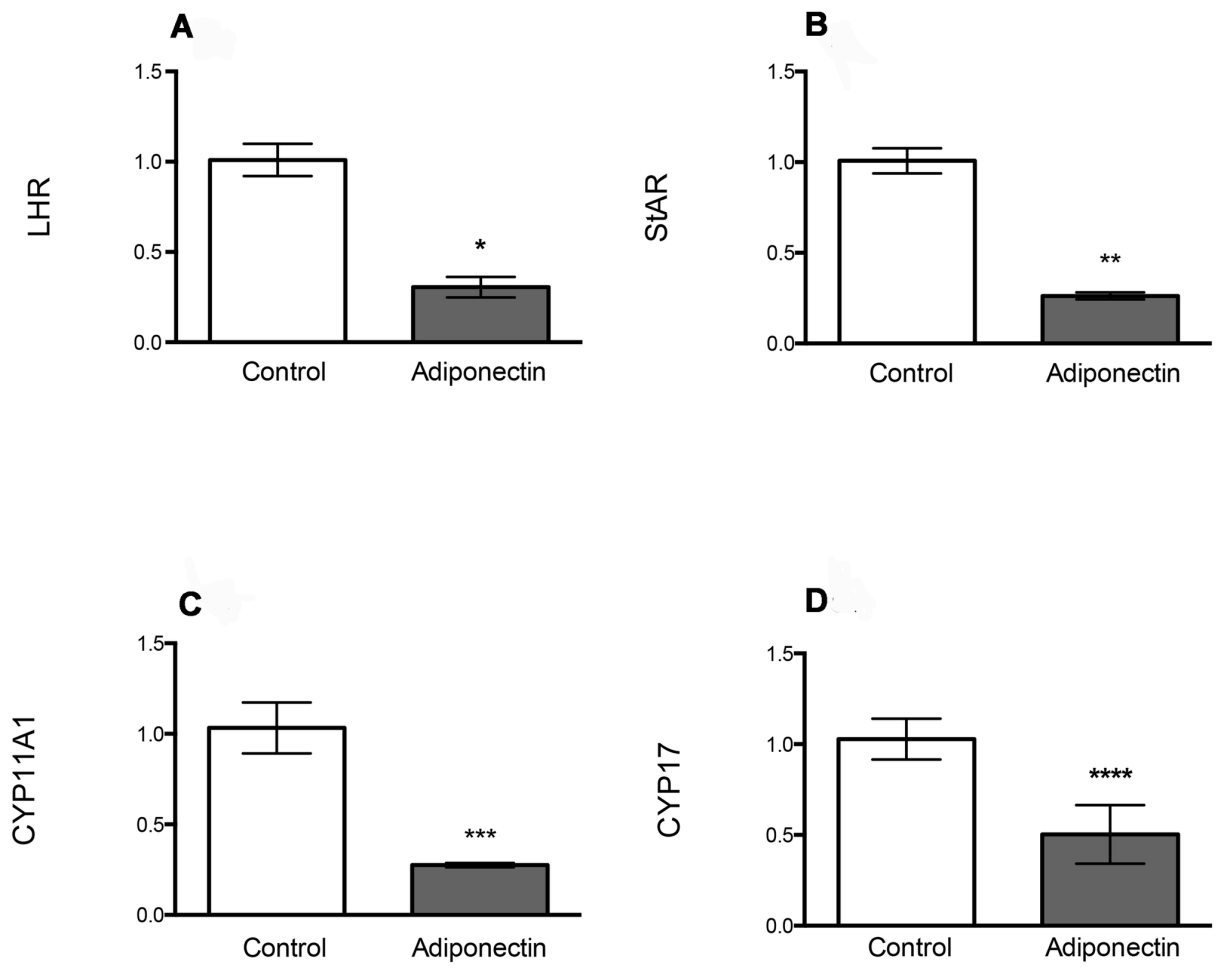
Gene expression of LH receptor (LHR), steroid acute regulatory protein (STAR) and key steroidogenic enzymes (CYP11A1, CYP17) after treatment with adiponectin. Cells were cultured in the presence of LH (10 ng/mL). The effect of adiponectin is expressed as a proportion of gene expression in control (non-treated) bovine theca cells. Marked decreases in the gene expression of (a) LHR (*p=0.02) (b), STAR (**p=0.028) (c) CYP11A1 (***p=0.02) and (d) CYP17 (****p=0.012) were observed in bovine theca cells after a 24-h treatment with adiponectin (3 μg/mL). Data are shown as the mean (95% CI).

In the presence of LH, adiponectin treatment not only reduced androstenedione levels in medium but also resulted in significant changes in gene expression of LHCGR and key steroidogenic enzymes in bovine TCs. As shown in Figure 3, after 24 h of treatment with adiponectin, there were significant decreases in the expression of LH receptor (LHCGR, 70%; p = 0.02), steroid acute regulatory protein (STAR, 74%; p = 0.028), P450 cholesterol side-chain cleavage (CYP11A, 72%; p = 0.012) and a key enzyme in androgen production, P450c17 (CYP17, 48%; p = 0.02).

### Suppression of adiponectin receptors results in enhanced androgen production

**Figure 4:**
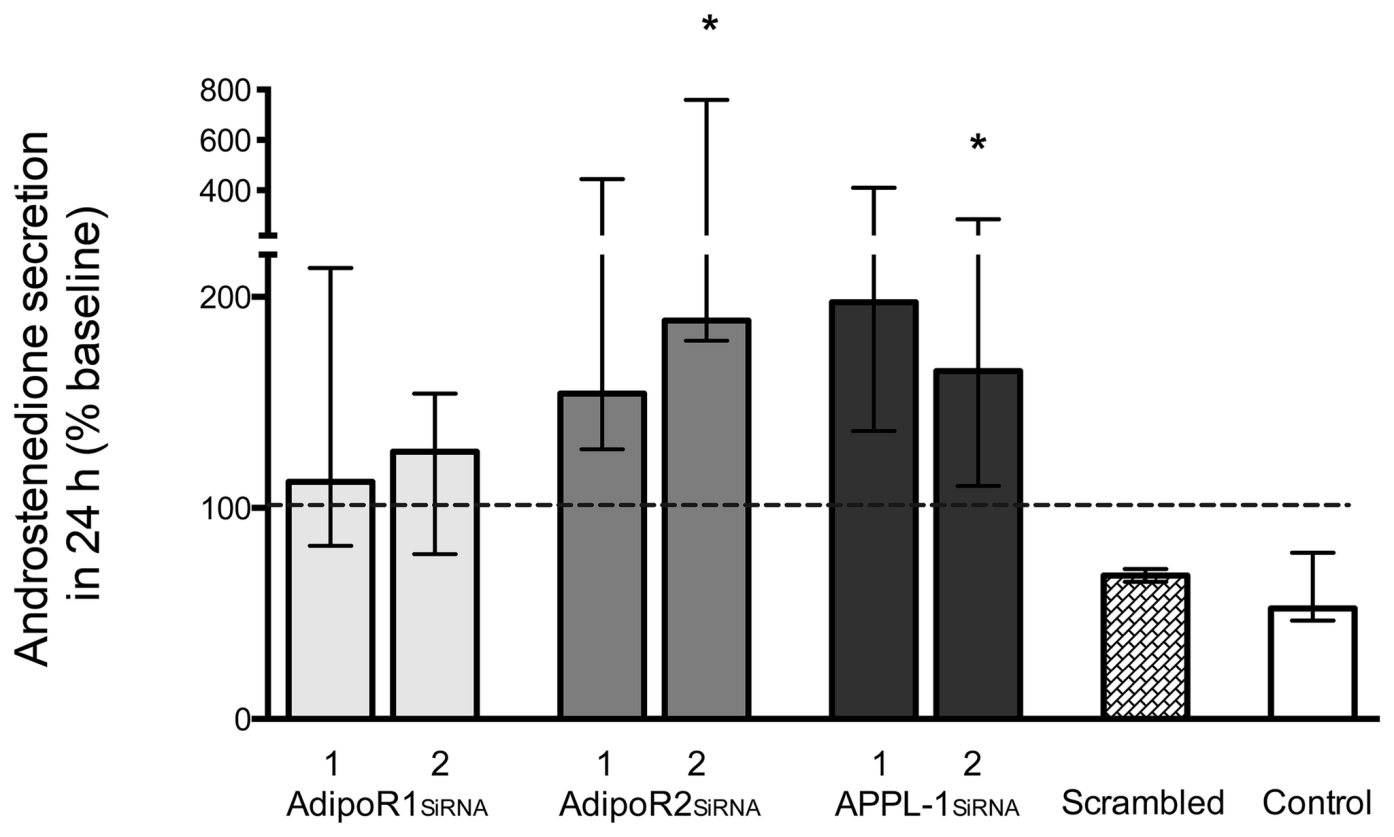
Effect of supression of adiponectin receptor gene expression on androstenedione production by bovine theca cells. Data are expressed as the change (%) from baseline values (0-hr; dotted horizontal line) after 24-h treatment with adiponectin plus LH (10ng/ml). SiRNAs were targeted against 2 separate oligonucleotide sequences (labeled 1 & 2) for each adipokine receptor and their common effector APPL-1 (AdipoR1, AdipoR2, or APPL-1). After 24-h treatment with adiponectin (3 μg/mL), a relative decrease in androstenedione secretion was observed in the control group (adiponectin alone) and the scrambled RNA group, whereas an increase over 100% (dotted line) was detected in all siRNA groups. Results are shown as medians and interquartile range. Overall changes as a result of siRNA treatments were significant (p=0.04 Kruskal Wallis) with significant changes indicated for knockdown of ADIPOR2 and APPL1 (*p=<0.05, Dunn’s mutiple comparison test vs control).

To address the role of adiponectin in regulating androgen production in TCs, knockdown of the genes encoding adiponectin receptors, AdipoR1, AdipoR2, and their common effector protein, APPL1, was carried out using specific siRNAs. As expected, TCs treated with adiponectin showed a decrease in androstenedione production: a reduction of about 40% compared with the basal level 24 h before treatment (Figure 4). A similar decrease (30% reduction) was observed in the presence of scrambled (control) siRNA. In contrast, specific gene knockdown produced increases in androstenedione secretion, reaching above 100% of the basal level: 119% to 136% of basal (adiponectin treatment alone) for AdipoR1 siRNA, 170% to 266% for AdipoR2 siRNA, and 177% to 257% for APPL1 siRNA (Figure 4). Thus, inhibition of the function of adiponectin receptors in theca cells resulted in enhanced production of androgen secretion, which suggests that adiponectin has an important physiological role in limiting androgen production by the ovary.

## Discussion

Our study supports the existence of a novel link between metabolic and reproductive features in PCOS and, specifically, implicates reduced action of adiponectin in the classic phenotype of PCOS, i.e., excess androgen production. We demonstrate that adiponectin, the most abundant secreted adipokine in humans (and which is usually reduced in the blood of obese PCOS patients), caused a decrease of about 40% in androgen secretion in a theca cells. Adiponectin-treated bovine theca cells showed a marked reduction of gene expression of the associated key steroidogenic enzymes and proteins including CYP17, CYP11, LHCGR, and STAR, which is in agreement with initial reports by Lagaly et al.[20] We also confirmed the importance of adiponectin and its receptors in the synthesis of androgens by showing that knockdown of adiponectin receptor expression (AdipoR1 and AdipoR2) or the downstream effector protein APPL-1, led to an increase in secretion of androstenedione. Interestingly, the suppressive effects of adiponectin on androgen secretion were not overcome by hyperinsulinemia but were dependent on the presence of LH, which is consistent with our observations that its mechanism of action appears to involve regulation of the LH receptor in theca cells.

One of the strengths of this report was to use a well characterized archive of ovarian tissue, which has been used previously in studies defining molecules involved in abnormalities in follicle development in PCOS [26,27]. Although we were unable, in this study, to use isolated human theca cells, there are sufficient data in the literature to support the view that the bovine theca cell model is a useful alternative for studying the effects of adipokines on thecal steroidogenesis, considering the functional similarities in theca between these two mammal species [31,32]. Furthermore human theca cell lines may not be ideal given that they may not express LHCGR (e.g. HOTT cells) [33]. 

Polycystic ovary syndrome is a disorder characterized by ovarian hyperandrogenism which can be exacerbated by obesity [7,34]. Excessive insulin secretion in obese women with PCOS plays a part in theca cell androgen production but here we provide evidence that adipokines – hormones secreted by fat cells - have a direct effect on the theca cell. Circulating adiponectin levels are also reduced in women with PCOS but in this study we report, for the first time, that expression of adiponectin receptors in theca cells from polycystic ovaries are reduced in comparison to normal ovaries. Changes in the expression of adiponectin receptors have previously been described in the fat tissue of women with PCOS but the results have not been consistent. Tan et al. identified an increase in adiponectin receptors in omental and subcutaneous adipose tissue from obese PCOS patients [20] whereas the opposite result was observed in another study of omental fat from lean women with PCOS [35,36]. To date, it remains unclear in the literature whether these changes in adiponectin receptors represent a cause or a consequence of obesity or insulin resistance. Our study included tissue from subjects amongst whom the minority (39%) was overweight or obese so we cannot completely exclude an effect of obesity on the expression of adiponectin receptors. Despite this limitation we, were able to compare expression of AdipoR1 and AdipoR2 between lean and overweight/obese women (either in the overall group or in the PCO group alone) and found no significant effect of BMI on adiponectin receptor expression in theca. It should also be noted that a susceptibility to PCOS in an association with polymorphisms of ADIPOQ (adiponectin) has been reported [37] and although this observation remains to be confirmed, it does support the view that the influence of PCOS on adiponectin receptor expression and function is independent of the effect of obesity. 

In summary, our data suggest that defective action of adiponectin, as a result of the reduction both of adiponectin serum levels and an associated reduction in adiponectin receptors in theca cells, contributes significantly to the exacerbation of androgen excess in overweight or obese women with PCOS. In addition, other adipokines, including leptin and visfatin can weakly enhance basal androgen secretion in bovine TCs by mechanisms that are yet to be identified. Drugs that target adiponectin are being developed with management of metabolic and cardiovascular dysfunction in mind and this raises the prospect of therapeutic application in women with PCOS. In conclusion, we propose that the observed disruption of adiponectin and its receptors is a major mechanism that links metabolic and reproductive dysfunction in women with polycystic ovary syndrome. 

## Supporting Information

Figure S1 **Characteristics of the theca cells used in the experiments.** (A)Theca cells exhibited *in* *vitro* conformational changes after 72 h of culture (10x magnification). (B) Merged picture (DAPI + CYP17A1 antibody) showing an imunofluorescent labelling in the cytoplasm. (C) Nuclear counterstaining with DAPI in the absence of antibody, (D)image of the negative control. In order to confirm the purity of theca cells, identification of molecular expression of CYP17A1 and exclusion of FSHR was also performed.
(TIF)

Table S1 **Clinical details of subjects for ADIPOR1 and ADIPOR2 immunohistochemistry.**
(PDF)

Table S2 **List of oligo sequences used for SiRNA.**
(PDF)

Table S3 **List of the primers used in the experiments.**
(PDF)
