# Supplementary figures and images for: Adiponectin and Its Receptors in the Ovary: Further Evidence for a Link between Obesity and Hyperandrogenism in Polycystic Ovary Syndrome
Source: PLoS One. 2013 Nov 18;8(11):e80416. doi: 10.1371/journal.pone.0080416 (PMC3832407; doi:10.1371/journal.pone.0080416)

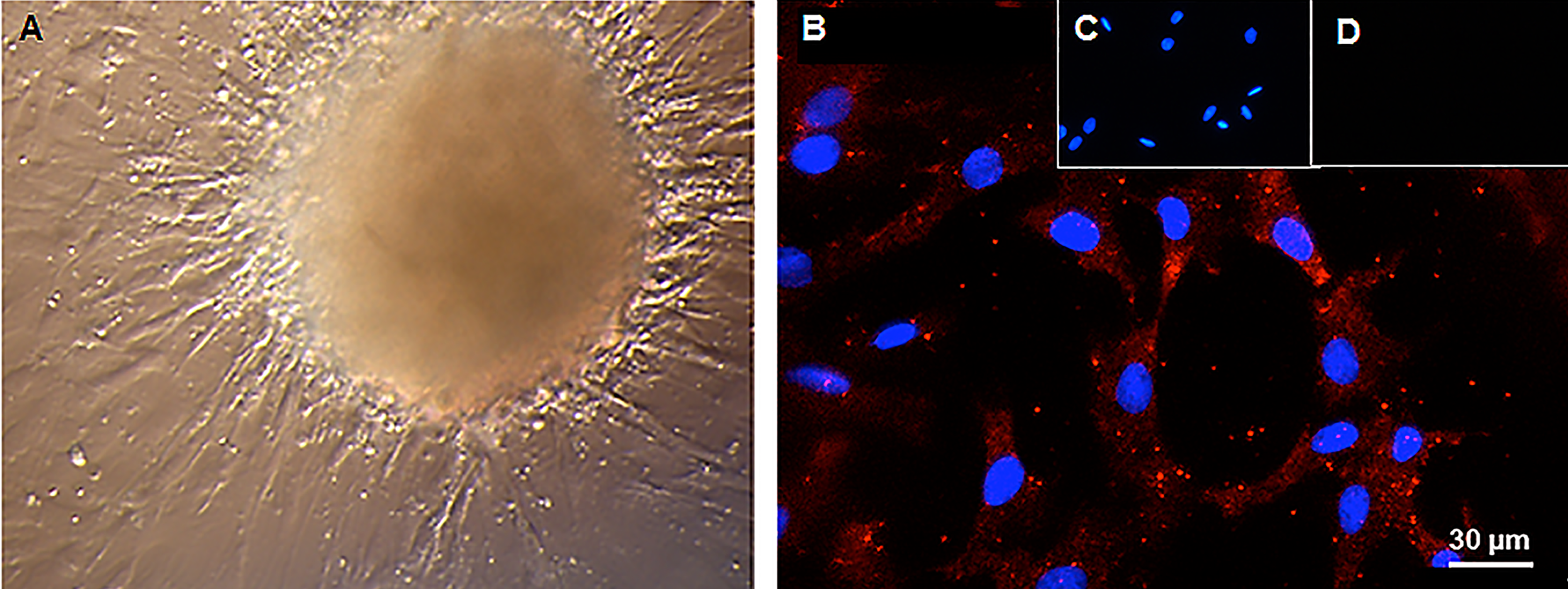

Supplement: Figure S1 — Characteristics of the theca cells used in the experiments. (A)Theca cells exhibited in vitro conformational changes after 72 h of culture (10x magnification). (B) Merged picture (DAPI + CYP17A1 antibody) showing an imunofluorescent labelling in the cytoplasm. (C) Nuclear counterstaining with DAPI in the absence of antibody, (D)image of the negative control. In order to confirm the purity of theca cells, identification of molecular expression of CYP17A1 and exclusion of FSHR was also performed. (TIF) [file pone.0080416.s001.tif]
