# Supplementary material for: Adiponectin and Its Receptors in the Ovary: Further Evidence for a Link between Obesity and Hyperandrogenism in Polycystic Ovary Syndrome
Source: PLoS One. 2013 Nov 18;8(11):e80416. doi: 10.1371/journal.pone.0080416 (PMC3832407; doi:10.1371/journal.pone.0080416)
Supplement: Table S1 — Clinical details of subjects for ADIPOR1 and ADIPOR2 immunohistochemistry. (PDF) [file pone.0080416.s002.pdf]

Table S1. Clinical details of subjects for ADIPOR1 and ADIPOR2 immunohistochemistry

Classification	Code	Age (years)	BMI (kg/m^2)	Ultrasound	Ovarian vol. (ml)	Menstrual cycle history[a]
**Normal**	A	26	27	normal	2.5	regular (4/28)
**Normal**	B	30	-	-	-	normal histology[b]
**Normal**	E	33	-	normal	-	regular (4/28)
**Normal**	Ja	38	-	-	-	normal histology[c]
**Normal**	F	33	17.5	normal	6.4	regular (5-7/21, prev regular 5/28)
**Normal**	H	34	20.2	normal	4.4	regular (2/28)
**Normal**	G	34	23.9	normal	-	regular (7/30)
**Normal**	I	38	24.2	normal	8.5	regular (5-10/26-33)
**Normal**	D	33	-	-	-	normal histology[d]
**OvPCO**	J	24	-	-	-	PCO + corpora albicans on histology[e]
**OvPCO**	K	30	19.3	PCO	9.6	regular (5/28)
**OvPCO**	L	30	24.8	PCO	-	regular (5/28-31)
**OvPCO**	M	31	-	-	-	PCO + corpus luteum on histology[f]
**OvPCO**	N	33	26.8	PCO	9.2	regular (7/28)
**OvPCO**	O	33	21.3	PCO	10	regular (5/28-31)
**OvPCO**	P	34	26.8	PCO	9.3	regular (7/28)
**OvPCO**	Q	34	29.4	PCO	7.3	regular
**OvPCO**	R	35	23.4	PCO	12.6	regular (7/33)
**OvPCO**	S	38	21.9	PCO	12	regular (7/32-35)
**OvPCO**	W	35	18.4	PCO	14.5	regular (5-6/30-32)
**AnovPCO**	T	44	-	PCO	8.5	irregular(33-42)
**AnovPCO**	U	30	38.5	PCO	10.3	irregular(2/52, 3/12)
**AnovPCO**	V	33	36	PCO	9.9	oligomenorrhoea (3wk-7m)
**AnovPCO**	X	35	21	PCO	14.1	oligomenorrhoea (7-9 wk)
**AnovPCO**	Y	40	40.9	PCO	-	irregular(4-11 wk)

[a]days of bleeding/cycle length. [b-f] no cycle history or ultrasound available; classified according to histology alone: [b,-d] normal, [e and f] PCO with evidence of recent ovulation.
