# Supplementary material for: Adiponectin and Its Receptors in the Ovary: Further Evidence for a Link between Obesity and Hyperandrogenism in Polycystic Ovary Syndrome
Source: PLoS One. 2013 Nov 18;8(11):e80416. doi: 10.1371/journal.pone.0080416 (PMC3832407; doi:10.1371/journal.pone.0080416)
Supplement: Table S2 — List of oligo sequences used for SiRNA. (PDF) [file pone.0080416.s003.pdf]

Table S2. List of oligo sequences used for SiRNA

	Sense	Antisense	Length	Nanomoles
**1) ADIPOR1**	CGGACUGGCUGAAGGACAAUU	UUGUCCUUCAGCCAGUCCGUU	21	20
**2) ADIPOR1**	UGGAGAAGAUGGAGGAGUUUU	AACUCCUCCAUCUUCUCCAUU	21	20
**3) ADIPOR2**	UGGAAGAGUUUGUUUGUAAUU	UUACAAACAAACUCUUCCAUU	21	20
**4) ADIPOR2**	GGAGAGGAUGGAAGAGUUUUU	AAACUCUUCCAUCCUCUCCUU	21	20
**5) APPL1**	GGAAAUGGACAGUGAUGUAUU	UACAUCACUGUCCAUUUCCUU	21	20
**6) APPL1**	GGAAAUUGCUGCACGAGUAUU	UACUCGUCCAGCAAUUUCCUU	21	20
