# Supplementary material for: Adiponectin and Its Receptors in the Ovary: Further Evidence for a Link between Obesity and Hyperandrogenism in Polycystic Ovary Syndrome
Source: PLoS One. 2013 Nov 18;8(11):e80416. doi: 10.1371/journal.pone.0080416 (PMC3832407; doi:10.1371/journal.pone.0080416)
Supplement: Table S3 — List of the primers used in the experiments. (PDF) [file pone.0080416.s004.pdf]

Table S3. List of the primers used in the experiments.

Target	Acession number	Forward sequence (5'-3')	Reward sequence (3'-5')	Species
**APPL1**	NM_001099003	AGCTGCTTCCAGTAGACCAAAC	TGCCATTCTACCACAGAAATTG	Bos taurus
**CYP11A1**	NM_176644	TGATGCCGTCTACAAGATGTTC	CAGTAGAGGATGCCTGGGTAAT	Bos taurus
**STAR**	NM_174189	CTTGAGGAGGTCAAGAGGTCTG	TGGAAGAAGTACGGGAATCAGT	Bos taurus
**ADIPOR1**	NM_001034055	CCTGCTCGGTTTCGTGCTGTTT	GGCGATCCCTGAATAGTCCAGC	Bos taurus
**ADIPOR2**	NM_001040499	GGGAAGGTCGATGGCGAGTGAT	GCACAGGAAGAACACACAACCCAA	Bos taurus
**CYP17A1**	NM_174304	GTTGTGTTCAAACGCGAAAT	GCCCACGATCCACTTTATCAC	Bos taurus
**LHR**	NM_174381	CAATGTTGAAAGCACAGCAAG	CACAGGGATTGAAAGCATCT	Bos taurus
**FSHR**	NM_174061	GCGGCAAACCTCTGACCTTC	GGGAGCAAGTCACATCAACCA	Bos taurus
**RPL19**	NW_001495013	CCTGGATGAGGAGGATGAGA	TTGTCTGCCTTCAGCTTGTG	Bos taurus
